# Interactions between reproductive biology and microbiomes in wild animal species

**DOI:** 10.1186/s42523-021-00156-7

**Published:** 2021-12-23

**Authors:** Pierre Comizzoli, Michael L. Power, Sally L. Bornbusch, Carly R. Muletz-Wolz

**Affiliations:** Smithsonian Conservation Biology Institute, National Zoological Park, Veterinary Hospital MRC5502, PO Box 37012, Washington, DC 20013 USA

**Keywords:** Wild animal species, Reproduction, Microbiomes, Conservation breeding, In situ conservation

## Abstract

Many parts of the animal body harbor microbial communities, known as animal-associated microbiomes, that affect the regulation of physiological functions. Studies in human and animal models have demonstrated that the reproductive biology and such microbiomes also interact. However, this concept is poorly studied in wild animal species and little is known about the implications to fertility, parental/offspring health, and survival in natural habitats. The objective of this review is to (1) specify the interactions between animals’ reproductive biology, including reproductive signaling, pregnancy, and offspring development, and their microbiomes, with an emphasis on wild species and (2) identify important research gaps as well as areas for further studies. While microbiomes present in the reproductive tract play the most direct role, other bodily microbiomes may also contribute to facilitating reproduction. In fish, amphibians, reptiles, birds, and mammals, endogenous processes related to the host physiology and behavior (visual and olfactory reproductive signals, copulation) can both influence and be influenced by the structure and function of microbial communities. In addition, exposures to maternal microbiomes in mammals (through vagina, skin, and milk) shape the offspring microbiomes, which, in turn, affects health later in life. Importantly, for all wild animal species, host-associated microbiomes are also influenced by environmental variations. There is still limited literature on wild animals compared to the large body of research on model species and humans. However, the few studies in wild species clearly highlight the necessity of increased research in rare and endangered animals to optimize conservation efforts in situ and ex situ. Thus, the link between microbiomes and reproduction is an emerging and critical component in wild animal conservation.

## Introduction

Animal evolution has taken place in environments replete with microorganisms. With a few exceptions (in some insects, for example), every animal species is colonized and lives in close association with microbial partners that include viruses, bacteria and microscopic eukaryotes [1]. Animal biology cannot be understood without reference to the microbial communities that have evolved to live on and within hosts, usually referred to as the organism’s microbiomes. This has led researchers to propose that the amalgam of host and the microbial community genomes might be a unit of selection [2]. The term microbiome is generally used to refer to both the composition of a microbial community (also termed the microbiota) and the entire microbial genomic content of the community (also termed the metagenome). The expressed microbial genome (also termed the metatranscriptome) is the fundamental biological unit of importance but is more difficult and expensive to determine compared to identifying the microbiota by 16S rRNA gene sequencing. The present review will be using the term ‘microbiome’ to refer to the microbial community composition.

**Fig. 1:**
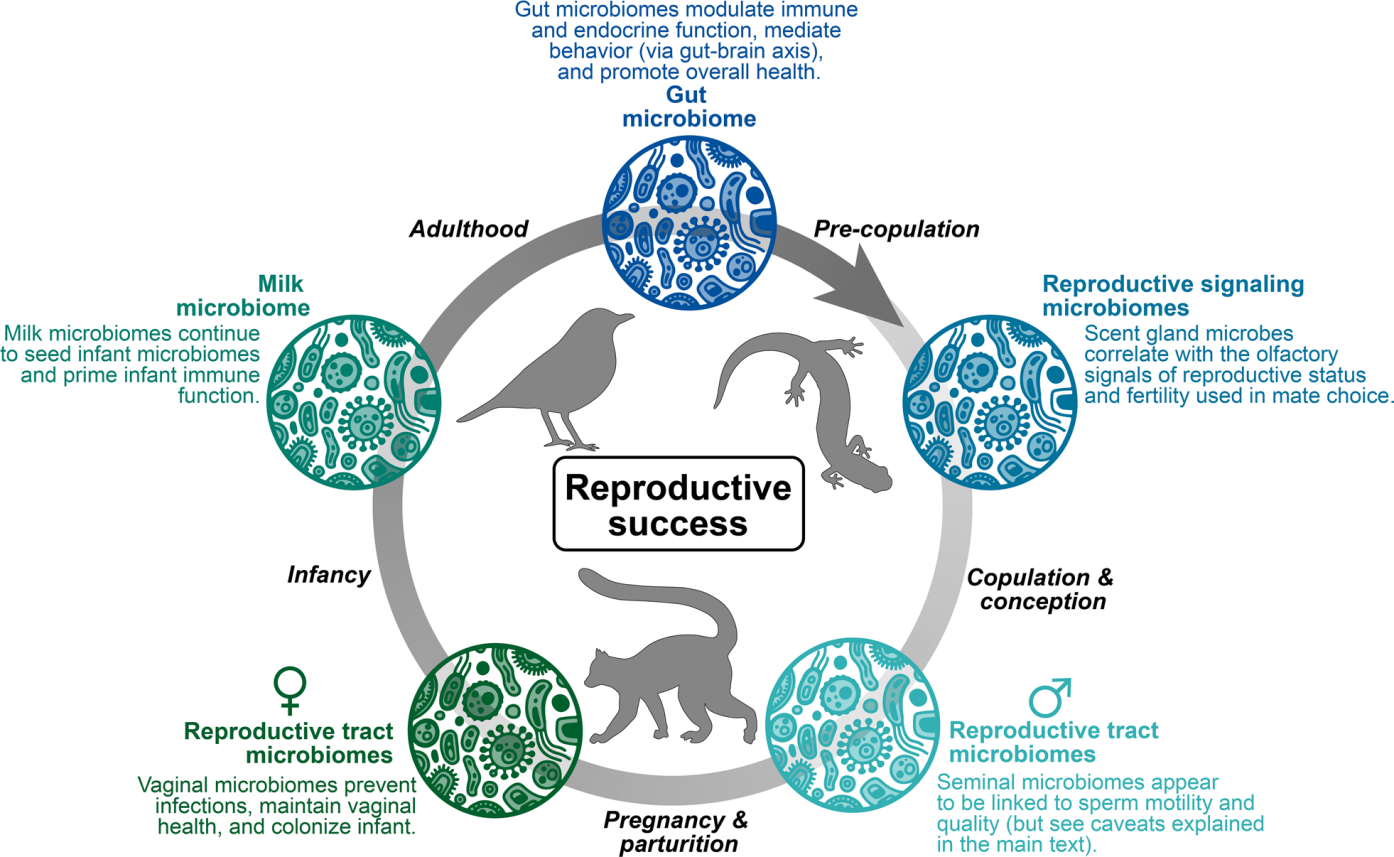
interactions between host microbiomes, life cycles, and reproductive success in wild animal species

The microbiome is a valuable proxy for estimating physiological states, including in reproduction, which is essential to the survival of species. Balanced interactions within and between host cells and non-host cells, such as the resident microbial community, are essential to overall fitness, including reproductive health. Reproductive microbiomes are defined as microbial communities inhabiting host reproductive tracts in males and females [3, 4]. However, other microbial communities (e.g., gut, milk, and glandular microbiomes) can further influence reproductive success from mate choice to healthy pregnancy and successfully producing offspring [2]. For example, signal-producing microbiomes (in e.g., scent glands) may play a role in pre-copulatory reproductive communication. Similarly, the milk microbiome influences post-natal infant development. Host-associated microbiomes, including reproductive microbiomes, are also affected by both endogenous processes, such as host physiology [5, 6] and exogenous factors, such as social interactions and environmental variations [7, 8]. Thus, while microbiomes present in the reproductive tract may play the most direct role, other bodily microbiomes may contribute to facilitating reproduction. Figure 1 illustrates the complex interactions between host microbiomes, life cycles, and reproductive success. It is recommended that research on reproductive microbiomes should also be expanded to include microbial effects before copulation/fertilization and the transfer of appropriate microbiomes to the offspring.

Some of the earliest observations on the linkages between host microbiota and host reproduction occur in freshwater hydra, in which bacteria are essential to the production of offspring through asexual budding [9]. Since then, a growing body of literature on the role of microbiota in host reproduction has been produced, with a large focus on invertebrate systems (particularly *Wolbachia* symbioses) and vertebrate model organisms [10]. This is in part due to the ability to perform controlled laboratory experiments in these systems that allow for inference regarding host reproductive-microbiota interactions. Not surprisingly, we know little about microbiomes in animal species compared to human microbiomes. Most reports on the reproductive effects of microbiomes of non-human species are on domesticated and laboratory animals, mostly mammals [3].

Although the number of microbiome studies conducted on wildlife remains lower than in other animal species, it has increased over the last decade [3]. Pronounced animal species declines and loss of natural habitats across the globe have led to the establishment of many captive assurance populations in which reproductive success is critical to the persistence of rare and endangered vertebrate animals [11]. Captive breeding is known to alter host-associated communities and there is increasing support for an associated effect on host reproductive success [12]. As observed in humans [13, 14], assisted reproductive technologies (from hormonal stimulation to embryo transfer), contraceptive methods, and treatment of pathologies may have an impact on the microbiomes. Our attempts to breed species in captivity (naturally or through assisted reproductive technologies) may have significant effects on the species microbiomes that will influence success or failure of reproduction.

The objective of this review is to (1) specify the interactions between animals’ reproductive biology, including reproductive signaling, pregnancy, and offspring development, and their microbiomes, with an emphasis on wild species and (2) identify important research gaps as well as areas for further studies. We will begin by discussing the links between microbiomes, life stage, and reproductive status in males and females. Then, we will move to detailing the link between microbiomes, reproduction, and environmental changes. We will end by discussing future directions in that area. Throughout the manuscript we also iterate the importance of including appropriate environmental controls and experimental criteria to improve the validity of reproductive microbiome research, since these are typically low biomass [15, 16].

## Links between microbiomes, life stage, and reproductive status in males and females

### Endocrine mediation of reproductive microbiomes across reproductive cycles or breeding seasons

The host endocrine system affects the structure of reproductive microbiomes in many animal species [5, 6], particularly through corticosteroid hormones (glucocorticoids and mineralocorticoids) [17, 18]. Glucocorticoids, such as cortisol and corticosterone, are associated with stress and can affect reproductive functions through known links to reproductive behavior and endocrine cycling as seen in primates, rhinos, and wild birds [6, 19–23]. [8]Evidence for bidirectional signaling between the microbiome and the hypothalamic pituitary axis and greater gut-brain axis also suggests that microbiomes can have significant effects on the host endocrine system [6]. Glucocorticoid-related microbiome effects have largely been studied in the gut microbiome with some investigation of reproductive outcomes in rhinos [23], fish [24], birds [22, 25], and pigs [26]. For example, in Eastern black rhinos (*Diceros bicornis michaeli*), glucocorticoid and progestagen concentrations, as well as the abundance of certain less common gut microbes, differ between female rhinos who produce calves and females that are considered non-breeders [23]. This suggests a relationship between hormone cycling, reproduction, and gut microbiome; however, the direction of these effects is unknown [23]. Bacteria can metabolize steroid hormones such as glucocorticoids and convert them into androgen hormones in humans [27], highlighting the challenge of disentangling hormone origin and the cause-and-effect relationships between microbiomes and reproduction.

Studies in humans also indicate that increased cortisol associated with prolonged stress can influence the vaginal mucosa, including the deposition of glycogen, which mediates the abundance of *Lactobacillus* spp. and underpins aspects of human vaginal tract health and copulatory success [28]. More broadly, increased cortisol resulting from prolonged stress is correlated with changes in gut microbiome structure and inflammatory responses, which, in turn, are known to affect pregnancy outcomes in women [29]. Although more research is needed in wild animal species, the relationships between glucocorticoid hormones and microbiomes provide plausible mechanisms by which psycho-social and physiological stress can influence health and reproductive outcomes.

Likewise, sex hormones play an integral part in structuring reproductive microbiomes. Previous research has largely been focused on female mammalian microbiomes and their relationships with ovarian hormones (estradiol and progesterone), reproductive cyclicity, or general variation in vaginal microbiomes, such as in non-human primates [7] and giant pandas (*Ailuropoda melanoleuca*) [30]. In primates, the increased circulating estradiol associated with sexual maturity in female mammals is linked to changes in vaginal epithelium function (e.g., increased glycogen production) and microbial community structure, further highlighting the complex relationships between endocrine function, host physiology, and microbial communities [7]. In mice models, wild-type and estrogen receptor knock-out mice even indicate an effect of estrogen binding on microbiome structure [31].

In males, testosterone concentrations are linked to seminal microbiome structure, sperm characteristics (see below), and infertility [32–34]. However, those studies still need to be confirmed with appropriate controls to discount the influence of DNA contamination on the results as seen in research on other microbiomes. In male birds, cloacal microbial diversity is positively correlated with testosterone levels, which in turn may affect rates of reproductive behaviors—such as extra-pair copulations—that increase opportunities for microbial transmission [35]. In free-living rufous-collared sparrows (*Zonotrichia capensis*), cloacal bacterial communities differ between the sexes when they are in breeding condition. In males, but not in females, the bacterial community becomes more diverse with the onset of reproduction, and then decreases in diversity as males transition to non-breeding condition [36].

Interactions between sex hormones and microbes can have significant metabolic effects on the host. In female Phayre’s leaf monkeys (*Trachypithecus phayrei*) reproductive hormones, specifically progestagens, contribute to the shifts in the gut microbiome during pregnancy and lactation [37]. There also is a shift in the maternal gut microbiome of Tibetan antelope (*Pantholops hodgsonii*) during the perinatal period [38], though the effects on reproduction are unknown. Future research over the next decade will likely elucidate how host endocrine-microbiome interactions affect host physiology and reproduction. At present, evidence for sex hormone-microbiome relationships in both sexes largely stem from studies of humans, whose reproductive microbiomes differ drastically even from other closely related primates (e.g., the unique *Lactobacillus* dominance in many, but not all, human vaginal microbiomes; [39]). The extent to which these patterns of endocrine-microbiome relationships apply to endangered species warrants further investigation, especially given the common use of husbandry practices that alter endocrine function (e.g., hormonal contraceptives; see below).

### Microbiomes and reproductive signaling (visual and olfactory)

Bodily microbiomes could potentially contribute to reproduction by mediating pre-mating reproductive signaling. Bodily microbiomes could potentially contribute to reproduction by mediating pre-mating reproductive signaling (e.g., plumage or bill color in birds [2, 40]). The strongest examples for reproductive signaling mediated by the microbiome stems from studies on olfaction. Olfactory signaling is a mode of reproductive communication that has long been thought to rely on a combination of volatile organic compounds produced by the host and by their commensal microbes (i.e., the fermentation hypothesis; [41]). Olfactory chemical cues can facilitate mate choice and reproductive success through ‘honest’ signaling of animal health, kinship and genetic compatibility, social status, and reproductive cycle and fertility [42–44]. Depending on the social organization of the animal species, these cues are relevant to and can be produced by one or both sexes. Although much of the previous research has been focused on male odor production to attract female mates, females of many species (including primates that are female-dominant) also rely on scent signaling to convey socially relevant information [45]. These cues can emanate from multiple body sites (e.g., scent gland and skin surfaces) and waste products (e.g., urine and feces) [46]. Scent glands, in particular, provide protected, ideal habitats for microbial growth and specialized glandular tissue can occur at various body sites, including homologous sites in males and females (e.g., labial glands in females and scrotal glands in males).

In multiple mammals, scent gland signals covary with glandular bacterial communities [47, 48]. These olfactory signals convey social and reproductive information that may influence social status and mate choice (for example in giant pandas [49] or European badgers [50]). In male diademed sifakas (Propithecus diadema) sternal gland microbiomes and olfactory chemical signals vary according to the male’s breeding vs. non-breeding status [51]. Similarly, in Chinese musk deer (Moschus berezovskii), musk gland bacteria and musk chemical composition reflected breeding status as mated vs. unmated, an important signal for mate choice [52]. Thus, scent gland microbes are associated with the host’s ability to advertise reproductive information, which may affect their reproductive success. Outside of mammalian species, bacteria from the uropygial glands of multiple bird species produce volatile compounds that are strongly linked to reproductive success [53] and appear to correlate with major histocompatibility complex diversity and reproductive compatibility [54]. Additionally, in a common South American tree frog (Dendrobates sp.), sex-specific scents can be traced to a frog-specific skin bacterium that produces a class of compounds with strong odor properties [55]. However, investigations of microbial contributions to scent signaling are scarce, particularly in wild animals. If, as mounting evidence suggests, host-associated microbes underpin signals of reproductive status and mate choice, investigation of this microbe-olfaction-reproduction axis may be especially relevant to holistic perspectives of reproductive success.

### Influence of mating behavior and copulation on microbiomes

In female mammals, there is a complex vaginal microbiome, which is vital to many aspects of reproduction, including preparing the host for reproductive activity. The disruption of the vaginal microbial community can have severe consequences for health and reproductive success [56–58]. The vaginal microbiome has been studied in a number domesticated mammal species, non-human primates and the giant panda (reviewed in [59]). Vaginal microbiomes vary widely among host taxa, and closely related species will not necessarily have similar vaginal microbiomes [60]. A Lactobacillus dominated vaginal microbiome appears unique to humans [7, 60] and even within humans there are stable, healthy vaginal microbiomes with few Lactobacillus [61].

In male mammals, semen is a complex biological fluid that contains nutrients for the spermatozoa (fructose, glucose, amino acids, minerals) factors that affect sperm motility and potency, and has a characteristic pH [62–65]. Many of the characteristics that make semen an adaptive medium for sperm cells also are conducive to microbial organisms. Certainly, pathogens responsible for many sexually transmitted infections (STIs) live in seminal fluid. The existence of microbes in seminal fluid implies that a microbiome exists in the seminal vesicles and/or other parts of the male urogenital tract [31]. Most of the research on seminal fluid microbiomes has been done in humans and rodent biomedical models, and showed microbial-associated sperm dysfunction [2]. Evidence from mice suggests that the seminal fluid microbiome may also have beneficial effects on reproduction [31]. Studies in men have found associations between microbial taxa and sperm concentration, motility, and quality. Some microbes are associated with a reduction in sperm quality, whereas others are associated with higher quality sperm, suggesting that certain microbes may be indicators of sperm quality [32, 66]. A recent study conducted in peccaries (Pecari tajacu) also highlighted the relation between preputial microbiome and semen quality [67]. As mentioned earlier, more studies are warranted to discount the influence of DNA contamination on the results (as seen in research on other microbiomes). Furthermore, the seminal microbiome may also have long term effects on fetal development that may influence later health and reproductive success of offspring [31]. Male mice fed a high-fat diet produced male offspring with poorer sperm quality [68], suggesting that host metabolism has effects on semen and its microbiome [31]. It is unknown whether these two effects are causally related, but it raises the possibility that the seminal microbiome may have next generational effects on reproductive success.

Copulation is a necessary component of vertebrate reproduction and also presents an opportunity for the transmission and interactions of microbes between host males and females in both directions. The transfer of microbes by copulation—and the associated risk of perturbing the established microbiome or STIs—likely exerts selective pressures on male and female reproductive microbiomes [7, 39]. In non-human primates, peak sexual receptivity and copulation behavior coincide with increased vaginal microbial diversity [69, 70], which may defend against the increased risk of microbial transmission via copulation, including STIs [71]. In owl monkeys (Aotus sp.), unmated males and females differ from mated animals in preputial and vaginal microbiomes, respectively, which was a result of the transfer of microbes via copulation [72]. Whether the microbiome within semen can benefit the female partner is unknown. Although sexual microbial transfer is most widely studied from the perspective of STIs, copulation may also be an important vector for the transfer of benign or even beneficial microbes [56]. Certainly, the microbial community within seminal fluid will at least briefly inhabit the female reproductive tract after copulation and may potentially influence fertilization and perhaps even implantation. Evidence from human studies suggests that the seminal and vaginal microbiomes of consistent sexual partners exhibit similarities and that the vaginal microbiome changes slightly after intercourse [73]. More numerous or frequent copulations (as seen in promiscuous species) have been linked to greater vaginal or cloacal microbial diversity (in mice: [74]; in primates [60, 75]; in lizards [76]). Greater vaginal microbiome diversity in promiscuous species can aid in creating a community that is more resilient to perturbation or infection and, ultimately, may improve reproductive success [71]. However, in humans, promiscuity has been correlated with dysbiosis of vaginal microbiomes [77], suggesting that frequent copulations with different partners could also disrupt or destabilize the vaginal microbial community.

### Pregnancy and birth are major colonizing events of microbiomes

The lower mammalian female reproductive tract (vulva, vagina and cervix) harbors complex, dynamic microbiomes which vary by region, hormonal state, and reproductive condition [58, 78]. A lack of stability in the vaginal microbiome in early pregnancy, especially with any significant presence of pathogenic strains, is associated with pregnancy complications including preterm birth and spontaneous abortion [79]. In contrast to the lower tract, the upper mammalian female reproductive tract (uterus, fallopian tubes) is considered a protected body region, with the expectation that it will be difficult for microbes to travel from the lower into the upper reproductive tract and become established. This does not mean that microbes cannot migrate along the tract or colonize these regions, but the diversity and density of the microbes in the upper reproductive tract is generally sparse compared to other body regions in women [80]. The developing fetus, while not in a sterile environment, is in a protected environment, and thus exposed to a lower abundance and diversity of microbes.

There is consistent evidence for a uterine wall microbiome in several species, including humans, horses, cows and rhesus macaques (Macaca mulatta) [59, 81]. The uterine microbiome is distinct from and is less densely populated than the vaginal microbiome [59]. There is evidence in both humans and horses for an association between uterine microbiome structure and successful pregnancy outcome [59]. However, more research is required to investigate the robustness of and mechanisms underlying this association.

There is controversy regarding the extent and stability of the microbial communities found in the mammalian placenta. Note that most of the evidence for or against a placental/fetal microbiome again comes from human data. A placental microbial community has consistently been found in human pregnancies that are complicated by pre-eclampsia and preterm birth (reviewed in [82]) and microbial invasion of the placenta is considered a causal aspect for these pathologies; however, several studies also reported the existence of a placental microbiome during uncomplicated pregnancy [83, 84]. Studies also found evidence of microbial communities in amniotic fluid [85]. However, subsequent studies have challenged these results, finding no detectable microbiota in amniotic fluid from uncomplicated pregnancies with intact membranes [86, 87]. A recent systematic review concluded there was no evidence for a placental microbiome in uncomplicated human pregnancy [82]. While microbial communities may exist in the uterus, the placenta, and the amniotic sac, these sites are largely sterile in humans [82, 88]. A study of uterine, placental and fetal tissue from four rhesus macaque pregnancies delivered by Cesarean section at approximately 130 days of the normal 166-day gestation found a uterine wall microbiome, but no evidence of placental or fetal microbial communities [81]. Recent studies in horses found evidence for microbiomes in placenta, amniotic fluid and meconium, indicating early establishment of a microbiome within the fetal gut by microbes of placental origin (reviewed in [89]). However, these studies were done on material collected after parturition, the abundance of microbes was low and thus the possibility of contamination cannot be discounted. There is no evidence for any placental microbiome in cattle [90] and bacterial loads of amniotic fluid were no different from negative controls in calves delivered by Cesarean section [91]. However, there are detectable, albeit low abundance, microbes in calf meconium [91]. Thus, colonization of the placenta, amniotic sac, and fetus by maternal microbes may reflect pathology rather than adaptive function. An ascending intrauterine infection is a risk factor for preterm birth and is associated with both maternal and fetal inflammatory response [92]. Growing evidence suggests that the interactions between maternal reproductive tract microbiomes and maternal and fetal immune response are important factors in birth outcome [92]. Prenatal exposure to microbes happens in pregnancies complicated with certain pathologies, but whether microbial communities in placental tissue can inoculate the mammalian fetus before birth remains uncertain.

Regardless of the mode of transmission, early life microbiome may impact later reproduction, as shown in the mouse model [93]. The microbes acquired at birth can affect offspring health later in life (as reviewed by [94]), and health status can have subsequent effects on reproductive success, such as obesity [95]. When the fetus travels through the reproductive tract it will become inoculated with the maternal microbiome [96]. In humans, infants born vaginally vs. by Cesarean section will have different skin and gut microbiomes [97, 98]. For egg laying vertebrates there is less evidence, but both lizard and bird eggs appear to harbor bacterial communities inside the shell [99]. Presumably this inoculation occurs in the upper regions of the oviduct before the shell has formed. The extent to which any maternal microbes transferred after shell formation can persist in the nest and thus be available to inoculate hatchlings is unknown. Similarly, Bornean foam-nest breeding frogs deposit eggs within foam nests with the internal nest colonized by bacteria (McGrath et al. in review for this special issue). The vertical transmission of the maternal microbiome to offspring is a critical aspect of successful reproduction, and the microbiome of the female reproductive tract is the first exposure for eggs and fetuses.

### Post-partum microbiomes and infant development/reproductive success, from mother to child

In contrast with the often brief exposure to maternal vaginal microbiomes, infant exposure to the maternal skin and oral microbiomes will be chronic, occurring over the much longer time period of neonatal dependence, but also within the context of well-developed (though still developing) neonatal microbiomes that may be capable of resisting colonization by novel microbes. Maternal skin contact may be more important for certain taxa. For example, in southern hairy-nosed wombats (*Lasiorhinus latifrons*), pouches contain communities of microorganisms that are substantially altered by the host reproductive cycle. More investigations into the roles that pouch microorganisms may play in marsupial reproductive health and joey survival are warranted [100].

The neonate will also be exposed to a microbiome unique to mammals, the milk microbiome. Milk contains a microbial community that can vary among mammalian species [101]. The origin of the milk microbiome is not well established, but evidence from rodent models suggests that bacteria may be transported from the maternal gut to the mammary gland [102]. Compared to the vaginal microbiome the milk microbiome is depauperate, both in terms of species numbers and total amount of microbial cells. However, a breastfed baby is estimated to consume 10^5^ to 10^7^ bacteria each day [103]. Suggested biological functions for the milk microbiome include inoculation of the neonatal gut, activation of mammary immune systems and cells [58], and stimulation of the developing neonatal immune system [104]. Another potential function is to develop immune tolerance in the neonate to commensal and symbiotic maternal microbes. Note that the milk microbiome is not the only aspect of milk that influences the neonatal gut microbiome. Milk contains live maternal immune cells, maternal immune function molecules, antibiotic molecule such as lysozyme, and prebiotic oligosaccharides that are not metabolized by the neonate but rather by the neonate’s gut microbes [58]. The relative importance of the milk microbiome compared to these other aspects of milk are not known. The transfer of a gut microbiome from parent to offspring through feeding is not just a mammalian trait. Several avian taxa regurgitate food into their chicks and produce crop secretions, termed ‘crop milk’ that they feed to chicks. These secretions contain similar bioactive constituents to mammal milk and contain microbes [105, 106]. All these exposures to maternal microbiomes shape the offspring microbiomes, which, in turn, affects later health.

There also are extensive interactions between microbes, their products and the host immune system that are often symbiotic, not antagonistic. The lack of a microbiome can disrupt neonatal development and gnotobiotic rodent models display significant deficiencies in anatomy, physiology, metabolism and immune function [107]. The development of the neonatal immune system appears to be highly influenced by microbiome effects [107]. Captive breeding programs for endangered species need to consider how management procedures may affect early life microbiomes.

## Link between microbiomes, reproduction, and environmental changes

### Captivity, microbiomes, and fertility control

Often attributed to the loss of native or beneficial microbes, the effects of captivity vary across host species but are undeniably a vital aspect of conservation breeding [108]. Moreover, efforts to manage or improve reproduction in captive populations, via contraceptives or assisted reproduction, can shape reproductive microbiomes with largely unknown consequences [3]. Beyond the scope of captivity, human activity, including the alteration and contamination of natural environments, can influence animal microbiomes and affect reproductive success; however, empirical links between these anthropogenic influences and reproductive microbiomes are currently sparse. In the face of extensive and continuing human influence on natural ecosystems and their inhabitants, there has been a recent and urgent call for greater ‘microbiome conservation’ in order to characterize and preserve global microbial diversity [99, 109, 110]. Here, we reinforce this call and suggest that the study and conservation of reproductive microbiomes are particularly relevant to endangered or rare species in which managed breeding programs are vital resources for species survival.

Understanding the effects of captivity on host-associated microbiomes is crucial to maintaining and improving the care and management of endangered species [108]. The majority of research to date has focused on gut microbial communities [108, 111] with few studies examining captivity-induced changes in reproductive microbiomes [112]. Nevertheless, burgeoning evidence of ‘cross-talk’ between gut and reproductive microbiomes suggests that many gut-centered outcomes of captivity can have downstream effects on the suite of reproductive microbiomes mentioned above [113, 114]. The factors driving gut microbiome variation between captive and wild animals (e.g. dietary mismatches; [115]) may similarly influence reproductive microbiomes by altering host metabolism and dictating available resources. For instance, Williams et al. [12] found that in southern white rhinoceros (*Ceratotherium simum simum*) fertility varied significantly with phytoestrogen profiles, the abundance of several gut bacterial taxa, and microbially-derived phytoestrogen metabolites. This suggests that diet may drive transformation of dietary phytoestrogens by the gut microbiome which then impacts reproductive outcomes. Even pre-copulation, if the effects of captivity alter scent gland microbial communities similarly to the widely reported effects seen in the gut microbiomes, associated olfactory signals could be altered, resulting in discordant signals and a disruption of reproductive communication.

Medical treatments received by animals in captivity also have the potential to shape the structure and function of reproductive microbiomes [70, 108]. Antibiotic treatment has been strongly linked to dysbiotic microbial communities, and thus antibiotic use in captive endangered animals may structure reproductive microbiomes and influence associated outcomes. For example, antibiotic treatment in captive primates result in alterations of gut microbiomes, with potential long-term consequences for gut health [116]. There also is evidence in lactating women that prenatal antibiotic use changes the milk microbiome, by decreasing the prevalence of beneficial microbes and increasing the proportion of potential pathogens [117]. Although very little is known about the prevalence and impact of antibiotic resistance genes in reproductive microbiomes, they may pose a particular risk to endangered animals in captivity, where the efficacy of antibiotic treatment is paramount to successful care and husbandry.

Additionally, artificial fertility control, via e.g., contraceptives, may further influence the reproductive microbiomes of captive animals. Hormonal contraceptives supply artificial reproductive hormones (e.g., depot medroxyprogesterone acetate) that disrupt natural endocrine processes to minimize fertility and prevent pregnancy. In hormonally contracepted humans, endocrine mediation of microbial communities is altered resulting in changes in the composition of reproductive microbiomes [118, 119]; however, evidence for the effects of contraceptives on the microbiomes of non-human animals is scarce. In humans and non-human primates, hormonal contraception has been linked to the dampening or disruption of olfactory cues used for reproductive communication [120–122] potentially reflecting changes in odor-producing bacteria. Given the propensity for reproductive dysfunction in many captive, endangered species, greater study of reproductive microbiomes in these settings can shed light on potential mechanisms of dysbiosis and, in turn, provide opportunities to integrate microbial ecology into successful husbandry practices.

### Environmental contaminants, microbiomes, and reproductive health

Environmental contaminants, including herbicides, pesticides, and heavy metal pollutants, can cause adverse effects on reproduction in wildlife [123, 124] and change host microbiomes [125–127]. The adverse effects on reproduction may be mediated or modulated by changes in the host microbiome [128]. For instance, gut microbes can metabolize environmental chemicals and thus modulate toxicity for the host animal [129]. Likewise, one of the most commonly used herbicides, glyphosate, inhibits the biochemical pathway for aromatic acid synthesis which is found in numerous bacterial species and can subsequently alter host-associated microbiomes [126]. Overall, the interactions among reproduction, contaminants and microbiomes have rarely been examined. One study found that glyphosate-based herbicides altered gut microbiomes and reproductive hormone levels in Japanese quails (*Coturnix japonica*) in a long-term study, but did not directly affect reproduction in either sex (maturation, testis size or egg production) [130]. Future studies on the effects of environmental contaminants on reproductive microbiomes should consider that these interactions may be subtle or potentially delayed in order to fully understand the risks related to contaminant exposure [131].

In addition, man-made antibiotics that leach into natural ecosystems can alter both environmental and host-associated microbial communities and facilitate the spread of antibiotic resistance genes [132, 133]. In contrast to the effects of antibiotic treatment done under veterinary care, antibiotic contamination of the environment can exert selective, but often non-lethal pressure on microbes, allowing them to evolve or spread resistance genes and expand the ‘resistomes’ in natural environments. It is now well-known that antibiotic-resistant bacteria in environmental settings (e.g., water, soil, food items) can be picked up by animals and humans [134–136], where they can be incorporated into host-associated microbiomes and potentially pose significant health risks [133, 137]. Indeed, in wild and captive populations of endangered ring-tailed lemurs (*Lemur catta*), antibiotic resistance genes covaried between host gut microbiomes and environmental soil microbiomes, a pattern that also reflected anthropogenic use of antibiotics and environmental contamination [138].

### Pathogens, microbiomes, and reproductive health

There are well known proximate mechanisms by which pathogens can influence reproduction, usually negatively. When animals mount an immune response due to pathogenic infection, animals can increase, and maintain or decrease their reproductive investment. Such life-history tradeoffs in energy allocation between reproduction and fighting infection are fundamental to fitness. We can broadly consider pathogens as part of the host microbiome as they often colonize and live as part of the microbial community during infection. Most studies to date on reproduction-infection tradeoffs have examined this in the context of single pathogen infections or perceived infection (e.g., vaccination). Evidence shows that trade-offs between reproduction and fighting infection go in all directions, and likely reflect unique pressures, such as age of individuals in male crickets [139].

A commonly tested hypothesis is that an individual will increase reproductive investment when immuno-compromised [140]. The terminal investment hypothesis predicts that individuals should invest more in their present reproduction if they are less likely to survive, with infection and disease being a potential cue of diminishing reproductive value. A variety of studies provide support for the terminal investment hypothesis in reproductive efforts, including in frog species infected with a fungal pathogen that up-regulate gametogenesis [141], increase male calling behavior as well as mating success [142]. Likewise, similar support exists in bird species. For example, great tits (*Parus major*) infected with haemosporidian parasites have increased reproductive success [143], blue footed boobies (*Sula nebouxii*) with elicited immune response increase parental care [144], and vaccinated house sparrows (*Passer domesticus*) increased egg laying efforts [145]. On the other hand, mounting an immune response can lead to reduction in reproductive investment. Another intriguing hypothesis is the immuno-competence handicap hypothesis which predicts that only high-quality males can maintain high levels of testosterone and afford the physiological cost of hormone-derived immunosuppression, but similar to the terminal investment hypothesis data remains ambiguous to support immunocompetence handicap hypothesis [146]. Future work examining how the larger collective microbial community is involved in these tradeoffs would be useful.

## Conclusions—future directions

While microbiomes present in the reproductive tract play the most direct role, other bodily microbiomes may contribute to facilitating reproduction in wild animal species. Endogenous processes related to host physiology can both influence and be influenced by the structure and function of microbial communities. Importantly, exposures to maternal microbiomes (through the vagina, skin, and milk) shape the offspring microbiomes later in life (Fig. 1). In many cases, we do not know if shifts in the microbiome are the cause of changes in the host reproductive status or a by-product of physiological changes.

In addition, host-associated microbiomes are also influenced in wildlife by exogenous factors, such as social interactions, environmental variations, or ex situ breeding programs. However, much more research is warranted in that area in order to optimize conservation efforts. Although our review is biased towards mammals, as there currently is more information regarding reproductive microbiomes, many of the concepts discussed in this review (as well as the next steps proposed below) could be widely applied to other vertebrates.

It is important to remember that there is still a need for more comprehensive microbe taxonomy (for better identification) and more studies about microbial functions. It is critical to culture as many of these ‘reproductively relevant’ microbes as possible and use standardized 16S protocols so we can compare across studies and find shared microbes. As mentioned earlier, it also is essential to reduce contamination bias, including appropriate environmental controls and experimental criteria to improve the validity of low microbial biomass research [15, 16]. We also want to further highlight the importance of using adequate controls in sampling and sequencing reproductive microbiomes. To improve ‘credibility’ of reproductive microbiomes – (1) numerous negative control samples should be taken throughout sampling and throughout procedures in the laboratory to be used to identify potential contaminants post sequencing, such as by using the package ‘decontam’ available in R [147] and (2) use of positive mock bacterial community controls to verify distribution of communities are not overly skewed and to verify spurious sequences are not being generated during molecular procedures in preparing libraries for sequencing.

To fill the gaps of knowledge in wildlife reproduction and microbiomes, we propose a list of topics that should be explored to better understand and possibly manipulate microbiomes in wild animal species:

- Relationship between hormones, reproduction, and gut microbiome in males and females (including the impact of hormonal contraception or stimulations).
- Effect of puberty and aging on microbiomes [148, 149].
- Microbial contributions to scent signaling and reproduction.
- Role of male and female microbiomes on sperm competition during and after mating.
- Role of seminal microbiome on the reproductive success of offspring (transgenerational effect).
- Role of microbiome during conception, early embryo development in the oviduct, implantation, delayed implantation [150].
- Association between uterine microbiome structure and successful pregnancy outcomes (leading to the development of non-invasive microbial markers of early pregnancy and fetal health, prediction of infant health).
- Influence of maternal diet on maternal and fetal microbiomes during pregnancy.
- Extent and stability of the microbial communities found in the mammalian placenta.
- Investigations into the function of the pouch microbiomes in marsupials.
- Exploration of the amphibian cloacal and spermic urine microbiome.
- Microbiomes and reproduction in invertebrates (e.g., oysters, corals).
- Consequences of captive breeding management on early life microbiomes and future reproductive fitness.
- Prevalence and impact of antibiotic resistance genes in reproductive microbiomes and their potential risk to endangered animals in captivity).
- Links between infertility or reproductive issues and microbiomes.
- Impact of artificial insemination or embryo transfer on microbiomes during gestation (does artificial insemination provide the same microbial signals as copulation?). Effects of semen microbiomes on in vitro fertilization.
- Manipulating or bioengineering the microbiomes to enhance or suppress fertility [151].

Lastly, in situ conservation efforts could benefit from more studies on interactions between reproduction, contaminants, and microbiomes. This could be conducted on sentinel species that could reflect the status of reproductive health in wild species sharing the same habitat. Application of ecological theory and advancement of analytical tools would help to understand what is structuring microbiomes [152, 153]. Collective findings will inform strategies to improve reproductive health in conservation breeding or in the wild. It will also help to develop conservation policy or legislation to include microbiome assessments for translocation or reintroduction of wild animal species in their natural habitats.

## Data Availability

Not applicable.
